# Screening and Identification of ssDNA Aptamer for Human GP73

**DOI:** 10.1155/2015/610281

**Published:** 2015-10-25

**Authors:** Jingchun Du, Jianming Hong, Chun Xu, Yuanyuan Cai, Bo Xiang, Chengbo Zhou, Xia Xu

**Affiliations:** ^1^Kingmed College of Laboratory Medicine, Guangzhou Medical University, Guangzhou 510182, China; ^2^Department of Laboratory Medicine, The First Affiliated Hospital, Guangzhou Medical University, Guangzhou 510120, China

## Abstract

As one tumor marker of HCC, Golgi Protein 73 (GP73) is given more promise in the early diagnosis of HCC, and aptamers have been developed to compete with antibodies as biorecognition probes in different detection system. In this study, we utilized GP73 to screen specific ssDNA aptamers by SELEX technique. First, GP73 proteins were expressed and purified by prokaryotic expression system and Nickle ion affinity chromatography, respectively. At the same time, the immunogenicity of purified GP73 was confirmed by Western blotting. The enriched ssDNA library with high binding capacity for GP73 was obtained after ten rounds of SELEX. Then, thirty ssDNA aptamers were sequenced, in which two ssDNA aptamers with identical DNA sequence were confirmed, based on the alignment results, and designated as A10-2. Furthermore, the specific antibody could block the binding of A10-2 to GP73, and the specific binding of A10-2 to GP73 was also supported by the observation that several tumor cell lines exhibited variable expression level of GP73. Significantly, the identified aptamer A10-2 could distinguish normal and cancerous liver tissues. So, our results indicate that the aptamer A10-2 might be developed into one molecular probe to detect HCC from normal liver specimens.

## 1. Introduction

Hepatocellular carcinoma (HCC) is one of the most common and highly malignant tumors worldwide [1]. At present, alpha-fetoprotein (AFP) assay and ultrasonography are employed in screening for early stage HCC. However, the sensitivity and specificity of these screening methods remain a major hurdle in early diagnosis of HCC. Because of the lack of a method for early diagnosis of HCC, the 5-year survival rate is less than 5% [2–4]. Therefore it is urgently needed to develop new methods for early diagnosis of HCC.

Golgi protein-73 (GP 73) is a type II Golgi membrane protein, which is significantly increased in HCC [5–7]. More interestingly, the sensitivity and specificity of GP73 for diagnosis of HCC are higher than those of AFP, which makes it be a better biomarker for early diagnosis of HCC [8–10]. Currently, an ELISA method that utilizes GP73 antibody is available for measurement of serum GP73.

Aptamers are short single-strand oligonucleotides, which could be selected from random oligonucleotides library via systemic evolution of ligands by exponential enrichment (SELEX) technology. Importantly, aptamers bind target molecules with high affinity and selectivity [11, 12]. Unlike antibodies whose purity and specificity may vary among different preparations, aptamers can be easily synthesized and are extremely stable [13]. In addition, they could be easily labeled with fluorescent dyes or other reporters for diagnosis purpose [14].

Here, we screened the random oligonucleotides library for ssDNA aptamers against GP73 and identified several aptamers. We further characterized a selected aptamer and verified that it could recognize GP73 expressed in hepatic tissue.

## 2. Materials and Methods

### 2.1. Expression, Purification, and Identification of Recombinant Human GP73

The encoding sequence of Human GP73 was first amplified by PCR using specific primers (5′-CGG GAT CCA TGG GCT TGG GAA ACG GGC-3′ and 5′-GGA AGC TTG AGT GTA TGA TTC CG-3′). After gel purification, the PCR product was digested with BamH I and Hind III and ligated into vector pET-32a. The ligation product was transformed into DH5*α* and recombinant clones were picked up for verification using PCR and enzyme digestion. The pET-32a-GP73 plasmid was finally confirmed by DNA sequencing.

The pET-32a-GP73 vector was transformed into* E. coli* BL21 (DE3) and positive clones, obtained by ampicillin selection, were induced to express GP73 by isoprophyl *β*-D-1-thiogalactopyranoside (IPTG) (1 mM) under low temperature (25°C). The cell culture was harvested, resuspended in cold phosphate buffered saline (PBS) buffer, and lysed by sonication on ice with 10 sec on and 10 sec off intervals for a total of 25 cycles. The supernatant was collected by centrifugation at 10,000 ×g for 20 min at 4°C and then applied to a Ni^2+^-chelating chromatography column. The column was eluted by a stepwise gradient of PBS containing different concentrations of imidazole (from 20 to 500 mM). The eluant was collected and analyzed by SDS-PAGE. The fractions containing recombinant protein GP73 were dialysed against PBS buffer and concentrated by concentrator (Eppendorf, Hamburg, Germany).

### 2.2. Western Blotting

The immunogenicity of the recombinant GP73 was detected by Western blotting with specific anti-GP73 antibody (Abcam, Cambridge, MS, USA). The purified GP73 protein or control samples (host cell lysate) were diluted with Laemmli buffer and denatured at 100°C for 5 min and centrifuged at 12,000 rpm for 5 min at 4°C. Supernatant was recovered, separated on 10% SDS-PAGE, and transferred onto 0.45 *μ*m PVDF membrane (Millipore, Billerica, MA, USA). After blocking membrane with TBS-Tween-20 containing 5% nonfat milk for 1 hour at room temperature, the membrane was incubated with anti-human GP73 antibody overnight at 4°C. Then, the membrane was incubated with horseradish peroxidase- (HRP-) conjugated secondary antibody for 1 hour at room temperature and was detected by chemiluminescence signaling kit (Cell Signaling Technologies, Beverly, MA, USA).

### 2.3. SELEX Library and Primers

The SELEX library contains a 45-base central random sequence flanked by two invariant 20-nucleotide regions in each end (5′-ACG CTC GGA TGC CAC TAC AG-N45-CTC ATG GAC GTG CTG GTG AC-3′). The forward primer (5′-ACG CTC GGA TGC CAC TAC AG-3′) labeled with or without digoxigenin (Dig) and reverse primer (5′-GTC ACC AGC ACG TCC ATG AG-3′) labeled with or without biotin were used in PCR to obtain the unlabeled, single-labeled, or double-labeled amplification products. All primers were synthesized by Invitrogen Biotech Co., Ltd.

### 2.4. In Vitro Screening Procedure

The selection of aptamers against GP73 was performed using a SELEX technology. First, the purified GP73 or BSA was adsorbed onto the wells of 96-well plates. In detail, the plates were coated with 200 *μ*L/well of 2 *μ*g/mL of GP73 or BSA dissolved in binding buffer (20 mM HEPES, 120 mM NaCl, 5 mM KCl, 1 mM CaCl_2_, and 1 mM MgCl_2_, pH 7.3) and incubated overnight at 4°C. After washing for three times with the binding buffer, the incubated plates were blocked with 3% (w/v) BSA solution for 2 hours at 37°C, and unbound BSA was removed. Second, 10 *μ*M of the random ssDNA pool was prepared in 100 *μ*L of binding buffer and denatured for 5 min at 95°C, cooled for 5 min at 4°C, and then placed at room temperature for 5 min. The denatured ssDNA pool was added into the BSA coated wells and incubated for 40 min at 37°C to eliminate the sequences that were able to bind BSA. The unbound sequences were collected, added into the GP73-coated wells, and incubated for 60 min at 37°C. After incubation, the wells were washed 3 times with binding buffer containing 0.05% Tween-20 to remove unbound and weakly bound sequences. The remained sequences that bound GP73 were incubated in elution buffer (20 mM Tris-HCl, 4 mM GITC, and 1 mM DTT, pH 8.3) for 10 min at 80°C and then collected. The eluted oligonucleotides were purified by DNA purification kit (Tiangen, Beijing, China). The purified oligonucleotides were amplified by optimized PCR (1 cycle at 95°C for 5 min; 12 cycles at 95°C for 30 s, 50°C for 30 s, and 72°C for 30 s; 1 cycle at 72°C for 2 min), with unlabeled forward primer and biotin-labeled reverse primer. After amplification, the PCR products were denatured using 150 mM NaOH solution, and the unlabeled aptamer strands were separated using streptavidin-coated magnetic beads (Invitrogen, Carlsbad, CA, USA) and concentrated by ethanol precipitation. The separated aptamer strands were used as ssDNA pool for the next round of SELEX until the tenth round of screening was finished.

### 2.5. Enzyme-Linked Aptamer Sorbent Assay (ELASA)

ELASA was used to monitor the binding affinity between screened ssDNA aptamers and GP73 protein. First, Dig-labeled forward primer was used to prepare Dig-labeled aptamers by PCR at the end of the first, second, fourth, sixth, eighth, and tenth round of SELEX selection, respectively. Then 4 *μ*g of Dig-labeled aptamers was dissolved in binding buffer, prepared as above, and added into GP73-BSA coated or BSA coated 96-well microtiter plate and incubated for 60 min at 37°C. After incubation, the wells were washed three times with binding buffer containing 0.05% Tween-20 to remove unbound and weakly bound ssDNA aptamers. Afterwards, 200 *μ*L of a 1 : 10^4^ dilution of anti-Dig-ALP (alkaline phosphatase) antibody was added and allowed to react for 30 min at 37°C. Finally, after three-time washes with binding buffer containing 0.05% Tween-20, 200 *μ*L of freshly prepared PNPP solution (1 mg/mL) was added and the absorbance was measured at 405 nm after 30 min incubation (after quenching with 100 *μ*L/well of 1 M NaOH). The assays were performed in triplicate.

### 2.6. Aptamer Sequencing and Secondary Structure Prediction

After the tenth round of screening, the purified aptamer strands were amplified by PCR with unlabeled primers, and the PCR products were cloned into the pMD19-T vector (Clontech, Beijing, China). Thirty positive clones were picked up and sequenced. The similarity of these sequences was analyzed by sequence alignment software (Clustal). One sequence, which appeared twice among these aptamers, was designated as A10-2 and subjected to secondary structure prediction using the mFold software [15] at 37°C in 150 mM [Na^+^] and 1 mM [Mg^++^], a web-based server for DNA folding and hybridization predicting (http://unafold.rna.albany.edu/?q=mfold/DNA-Folding-Form).

### 2.7. Kinetics and Binding Capacity Studies of A10-2 for GP73

First, the recombinant GP73 protein or fetal bovine serum (FBS) was diluted to a series of concentrations (31.25 ng/mL–4 *μ*g/mL) in binding buffer and 200 *μ*L of each solution was incubated in a 96-well microtiter plate overnight at 4°C. After washing for three times with binding buffer, the incubated plates were blocked with 3% (w/v) BSA solution for 2 hours at 37°C and then again washed three times in order to completely remove unbound BSA. Dig-labeled A10-2 were synthesized in Invitrogen Biotech Co., Ltd., dissolved in binding buffer at 40 ng/*μ*L concentration and then denatured for 5 min at 95°C, cooled for 5 min at 4°C, and then placed at RT for 5 min. Afterwards, 200 *μ*L of A10-2 was added into each well and the plate was incubated at 37°C for 2 hours and washed three times. Then anti-Dig-ALP antibody was added and developed using PNPP solution as mentioned above. The change of absorbance at 405 nm was measured by a microplate reader (Thermo, Shanghai, China) and the assays were performed in triplicate.

Second, three different concentrations of GP73 protein (5 × 10^2^, 1 × 10^3^, and 2 × 10^3^ ng/mL) were incubated in the 96-well microtiter plate (200 *μ*L/well) overnight at 4°C, and BSA solution was used to block the plate as above. Afterwards, 200 *μ*L of Dig-labeled A10-2 was diluted into different concentrations (0.078–40 ng/*μ*L) in binding buffer and added into each well. The plate was incubated at 37°C for 2 hours, after which individual well was washed three times with binding buffer to remove unbound Dig-labeled A10-2. Then anti-Dig-ALP antibody was added and developed using PNPP solution as mentioned above. The change of absorbance at 405 nm was measured by a microplate reader (Thermo) and the assays were performed in triplicate.

### 2.8. Specificity Studies of A10-2 for GP73 by Antibody Blocking and Western Blotting Experiments

The recombinant protein GP73 was diluted into 2 *μ*g/mL in binding buffer and 200 *μ*L of solution was incubated in a 96-well microtiter plate overnight at 4°C. After washing for three times with binding buffer, the incubated plates were blocked with 3% (w/v) BSA solution for 2 hours at 37°C and again washed three times in order to completely remove unbound BSA. The anti-GP73 antibody (Abcam) was diluted into a series of concentrations (0.03125–1 *μ*g/mL) and 200 *μ*L of each solution was added into the GP73-BSA coated plate and incubated for 1 hour at 37°C; binding buffer without anti-GP73 antibody was used as negative control. Then the microtiter plate was washed for three times with binding buffer and further incubated with 200 *μ*L of Dig-labeled A10-2 (40 ng/*μ*L). The plate was incubated at 37°C for 2 hours, after which individual wells were washed three times with binding buffer to remove unbound A10-2. Then anti-Dig-ALP antibody was added and developed using PNPP solution as mentioned above. The change of absorbance at 405 nm was measured by a microplate reader (Thermo) and the assays were performed in triplicate.

To further confirm the specificity of A10-2 for GP73, the expression of GP73 in tumor cells HepG2, MCF-7, MDA-MB-231, MDA-MB-435, and A549 was measured by Western blotting, in which the biotin-labeled A10-2 (5 ng/*μ*L) and streptomycin-conjugated HRP functioned as primary antibody and second antibody.

### 2.9. Immunohistochemical Staining

Formalin-fixed and paraffin-embedded tissue blocks derived from 20 normal and hepatocellular carcinoma (HCC) tissues samples were obtained from the First Affiliated Hospital of Guangzhou Medical University with the approval of the Hospital Research Ethics Committee. For histochemistry staining, 5 *μ*m thick sections from tissue blocks were placed on microscopic slides and dried in a 60°C oven for 2 hours. Then, the sections were deparaffinized in xylene, rehydrated using gradient concentrations of ethanol, microwaved in 10 mM citrate buffer for 15 minutes to retrieve antigen, and blocked with 3% hydrogen peroxide for 10 minutes to inhibit endogenous peroxidase activity. After that, the sections were incubated with biotin-labeled A10-2 (40 ng/*μ*L, 1 : 500 dilution) at 4°C overnight and then incubated with streptavidin-conjugated HRP (Abcam) at RT for 1 hour. Then, color development was performed with a DAB Plus Kit (Maixin, Fuzhou, China). Finally, the sections were counterstained in hematoxylin.

### 2.10. Statistics

Data are presented as means ± SEMs from three to five independent measurements in separate experiments and analyzed using SPSS version 12.0 (SPSS Inc., Chicago, IL, USA). A *P* value of <0.05 was considered to be statistically significant.

## 3. Results

### 3.1. Expression, Purification, and Identification of Human GP73 Protein

**Figure 1:**
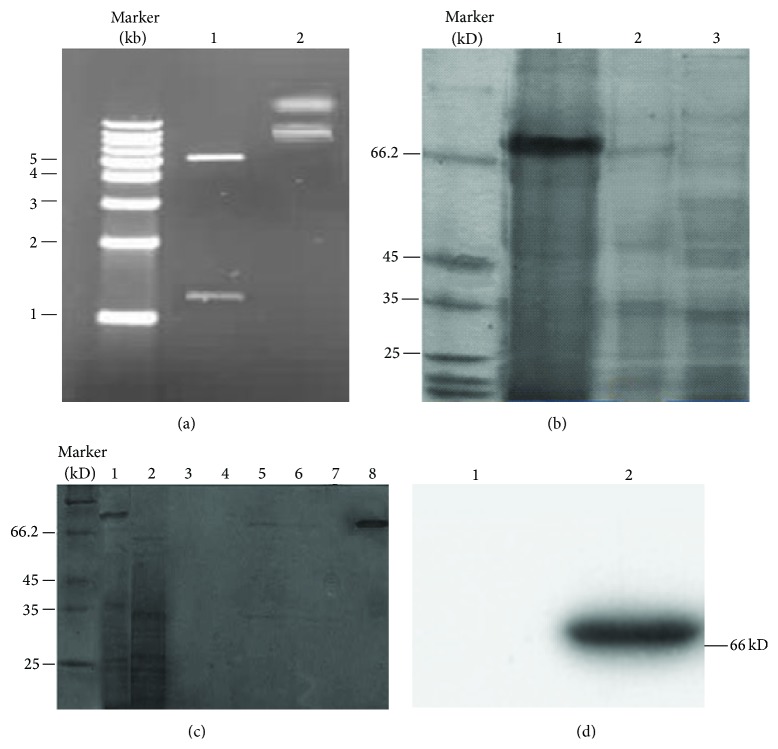
Preparation of the recombinant protein GP73. (a) The recombinant vector pET-32a-GP73 was confirmed by enzyme digestion. Lane 1, the double enzyme digestion product of the recombinant vector; Lane 2, the recombinant vector without double enzyme digestion. (b) The recombinant protein GP73 was induced in BL21 (DE3) by IPTG (1 mM) at 25°C. Lane 1, the supernatant of host BL21 (DE3) bearing pET-32a-GP73 with induction; Lane 2, the sediment of host BL21 (DE3) bearing pET-32a-GP73 with induction; Lane 3, the total lysate of BL21 (DE3) bearing pET-32a-GP73 without induction. (c) The recombinant protein GP73 was purified by affinity chromatography. Lane 1, the sample containing recombinant protein GP73 before loading affinity chromatography column; Lane 2, the eluate after loading affinity chromatography column; Lane 3, the eluate with PBS containing 20 mM imidazole; Lane 4, the eluate with PBS containing 40 mM imidazole; Lane 5, the eluate with PBS containing 60 mM imidazole; Lane 6, the eluate with PBS containing 80 mM imidazole; Lane 7, the eluate with PBS containing 100 mM imidazole; Lane 8, the eluate with PBS containing 500 mM imidazole. (d) The immunogenicity of the recombinant protein GP73 was confirmed by Western blotting with specific anti-GP73 antibody. Lane 1, the negative control; Lane 2, the purified GP73.

To prepare the recombinant human GP73 protein, the prokaryotic expression vector pET-32a-GP73 was constructed. As shown in Figure 1(a), the encoding sequence of GP73 was correctly inserted into the multiple cloning sites of pET-32a. After the pET-32a-GP73 plasmid was transformed into host* E. coli* BL21 (DE3), a single clone containing the expression vector was cultured into *A*_600_ = 0.6 and induced to express the recombinant protein GP73 with 1 mM IPTG at 25°C. The expression efficiency and intracellular solubility of GP73 were analyzed by SDS-PAGE. The target protein was expressed with a molecular weight of approximate 68 kDa, which was identical to the predicted size (Figure 1(b)).

The Nickel ion affinity chromatography was used to purify the target protein, because the recombinant GP73 was expressed as a fusion protein with His Tag. As shown in Figure 1(c), the target protein GP73 combined closely with Ni^2+^-saturated matrix was collected with above 90% purification after the nonspecific parts were eluted with a series of concentrations of imidazole solution in PBS buffer. At the same time, the immunogenicity of the recombinant human GP73 was assessed by Western blotting. As shown in Figure 1(d), the recombinant human GP73 was recognized by specific anti-GP73 antibody.

### 3.2. Selection of Specific Aptamers for Human GP73 Protein

**Figure 2:**
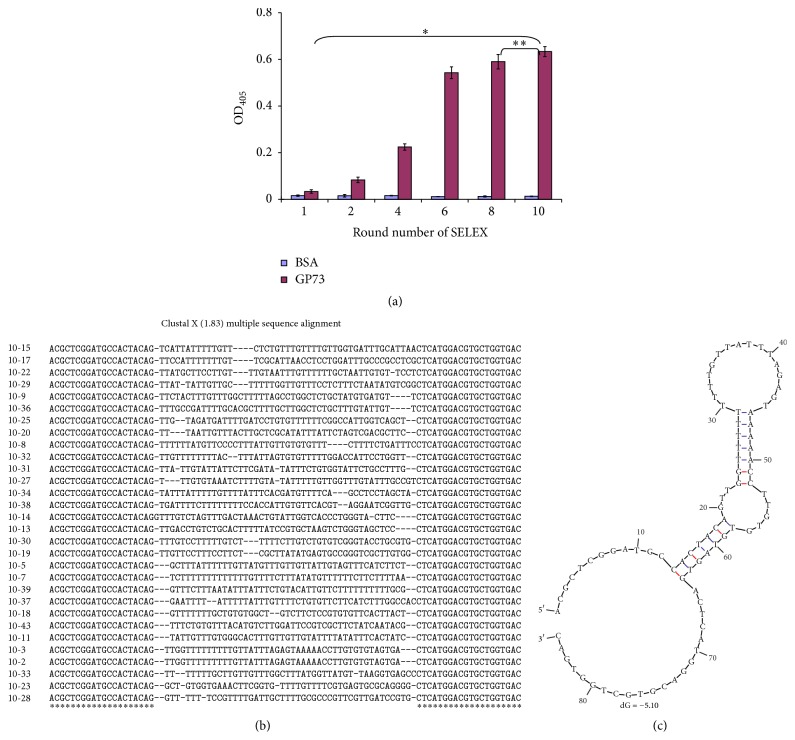
The SELEX screening of aptamers for GP73 and sequence analysis and structure prediction of aptamer A10-2. (a) The SELEX procession was monitored by ELASA. The binding capacity between selected ssDNA pool and GP73 was gradually increased with the increase of SELEX screening procession. (b) The encoding sequences of thirty aptamers derived from the tenth round of ssDNA pool were aligned and the sequences of two aptamers were the same, which were named A10-2. (c) The secondary structure and dG of A10-2 were analyzed using online mFold software. Asterisks indicate statistical significance: ^*^<0.01 and ^**^>0.05.

The aptamers specific for GP73 protein were selected by SELEX from an ssDNA library with a 45-nucleotide random region flanked by 20-nucleotide 5′ and 3′ fixed regions. The screening process was monitored by ELASA, which reflected the binding stringency between screened ssDNA library and target protein. As shown in Figure 2(a), the binding stringency between the ssDNA pool and GP73 gradually increased in small quantities before the fourth round (OD_405_ = 0.225 ± 0.014). However, the binding stringency rapidly increased after the sixth round (OD_405_ = 0.543 ± 0.025). There was no obvious difference in binding stringency between the eighth round (OD_405_ = 0.59 ± 0.031) and the tenth round (OD_405_ = 0.634 ± 0.022). The change of the binding stringency indicates that the ssDNA pool was enriched after the end of the screening process.

The ssDNA aptamer sequences, derived from the tenth enriched pool, were cloned into the pMD19-T plasmid, and thirty clones were picked for sequencing analysis. The results of sequence alignment showed that two clones were completely identical in nucleic acid sequence and the rate of reappearance was 6.7%, which was designated A10-2. The structure of aptamer A10-2 was predicted using one online program mFold, which yielded one potential secondary structure in the defined condition (Figure 2(c)). The aptamer A10-2 showed complex secondary structures, including protruding loops and stems. The Gibbs free energy (dG) of the aptamer was −5.1, suggesting that the aptamer A10-2 might maintain a rather stable structure.

### 3.3. Binding Capacity of Aptamer A10-2 to Human GP73 Protein

**Figure 3:**
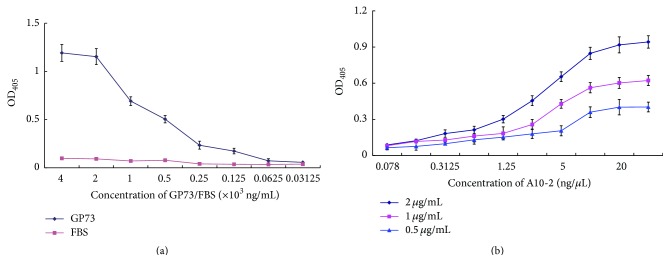
The binding capacity of A10-2 for GP73. (a) The recombinant protein GP73 was plated in a series of concentrations (31.25 ng/mL to 4 *μ*g/mL) and incubated with Dig-labeled A10-2. Finally, anti-Dig-ALP antibodies were added and revealed with PNPP solution at 405 nm. (b) Three concentrations of recombinant protein GP73 were plated and incubated with several concentrations (0.078–40 ng/*μ*L) of Dig-labeled A10-2. Afterwards, anti-Dig-ALP antibodies were added and revealed as above. The *K*_*d*_ of A10-2 was calculated by nonlinear regression analysis. All experiments were performed in triplicate and repeated at least for three times.

In order to determine the binding capacity of A10-2 for GP73, the appropriate concentrations of GP73 were first determined in an ELASA assay. A series of concentrations of recombinant protein GP73 (from 31.25 ng/mL to 4 *μ*g/mL) were absorbed in microplate wells and incubated with Dig-labeled A10-2 and the quantity of A10-2 specifically binding to GP73 was determined after incubation with anti-Dig-ALP antibody. As shown in Figure 3(a), the aptamer A10-2 bound GP73 in a protein concentration-dependent manner and the binding capacity almost reached a plateau at the concentration of 2 *μ*g/mL of GP73. As far as the concentration of A10-2 in our experiment was concerned, the binding capacity of A10-2 with GP73 changed most obviously in three consecutive concentrations of GP73 (2, 1, and 0.5 *μ*g/mL). In view of these results, the binding capacity of A10-2 for GP73 was determined under the condition of the three concentrations of GP73.

In order to determine the binding capacity of A10-2 for GP73 protein, the ELASA assay in which the 96-well plates were coated with three concentrations of GP73 protein was performed and a series of concentrations of Dig-labeled A10-2 were tested. Data were analyzed using nonlinear regression showing that they respond to a one-site binding curve with an equation *y* = (*x* × *B*_max_)/(*x* + *K*_*d*_), where *B*_max_ is the maximal binding and *K*_*d*_ is the concentration of ligand required to reach half-maximal binding. Data presented in Figure 3(b) indicated that A10-2 is able to detect GP73 protein in a concentration-dependent manner with *K*_*d*_ = 127.4 ± 18.65 nM.

### 3.4. Binding Specificity of Aptamer A10-2 for Human GP73 Protein

**Figure 4:**
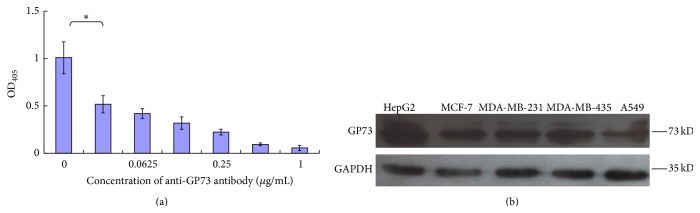
The specificity of A10-2 binding to GP73. (a) Before the Dig-labeled A10-2 was added and incubated with GP73-coated plate, the plate was first incubated with a series of concentrations of specific anti-GP73 antibody. Then the plate was continuously incubated with Dig-labeled A10-2 and revealed as above. (b) Biotin-labeled A10-2 recognized GP73 expressed in different tumor cell lines. All experiments were conducted in triplicate and repeated at least for three times. The asterisk indicates *P* < 0.01.

In order to determine the binding specificity of A10-2 to GP73, the specific anti-GP73 antibody was used to evaluate whether it could block the interaction between GP73 and A10-2. As shown in Figure 4(a), aptamer A10-2 could bind GP73 with high specificity while the binding capacity of A10-2 for GP73 dramatically declined when anti-GP73 antibody was first incubated with the coated GP73. At the same time, the binding capacity of A10-2 for GP73 was correspondingly decreased as anti-GP73 antibody concentration increased. In addition, A10-2 could replace anti-GP73 antibody to recognize GP73 protein in tumor cells lines (Figure 4(b)). These results indicate that aptamer A10-2 is specific for GP73.

### 3.5. Aptamer A10-2 Specially Recognizes GP73 in Human HCC Specimens

**Figure 5:**
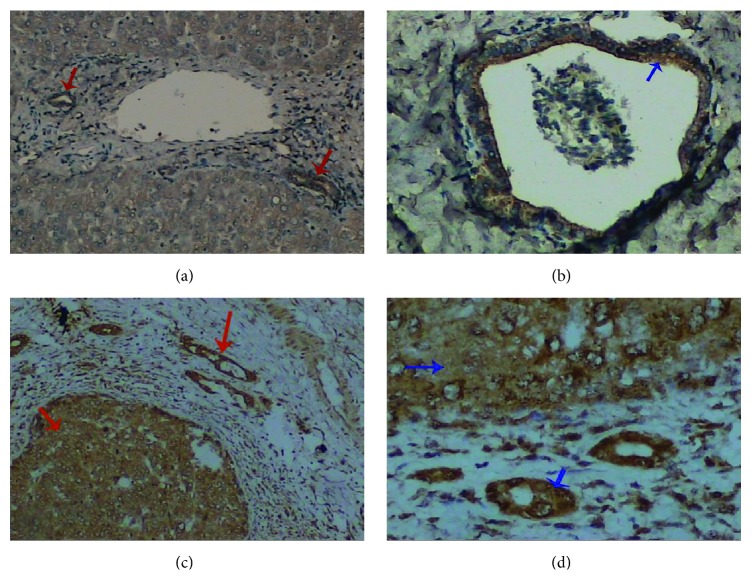
Detection of GP73 in human liver tissues by the aptamer A10-2. Tissue-specific GP73 expression was studied in normal liver and HCC tissues by indirect immunohistochemistry, in which Dig-labeled A10-2 were used as molecular probe. In normal tissue, GP73 is expressed in biliary epithelial cells ((a) and (b)). In HCC tissue, the hepatocytes and biliary epithelial cells were both strong stained ((c) and (d)). Arrows indicate the location of GP73. (a) and (c): original magnification 200x. (b) and (d): original magnification 400x. The results were the representations of 20 normal and HCC tissue samples.

In order to explore whether the A10-2 could be a potential probe to recognize tumor marker GP73 in vitro, we performed immunohistochemical staining in HCC specimens that had been pathologically confirmed. As shown in Figure 5, biotin-labeled A10-2 could clearly recognize GP73 in liver specimens. In normal liver tissue, GP73 was mainly located in the edge of liver bile duct. However, it was highly expressed in HCC tissue.

## 4. Discussion

At present, the curative treatments of HCC are mainly determined by whether HCC could be diagnosed in the early stage. Medical imaging exams and serum assay of alphafetoprotein (AFP) levels are the major ways to evaluate HCC. However, the sensitivity and specificity of AFP are not sufficient for clinical requirement and ultrasound examination could not find tumor with <1 cm diameter [16, 17]. As one new tumor marker of HCC, GP73 holds the promise for early diagnosis of HCC because the sensitivity and specificity of GP73 are both superior to those of AFP [18, 19].

In this study, we utilized GP73 as a target protein to screen specific ssDNA aptamers by SELEX technique. First, microgram levels of GP73 protein were expressed and purified by prokaryotic expression system and Nickel ion affinity chromatography, respectively. At the same time, the immunogenicity of purified GP73 was confirmed by the specific anti-GP73 antibody. The enriched ssDNA library with high binding capacity for GP73 was obtained after ten rounds of SELEX. Then, thirty ssDNA aptamers were sequenced and the nucleic acid sequences were aligned, in which two ssDNA aptamers with identical DNA sequence were confirmed, based on the alignment results, and designated as A10-2.

In the last decade, aptamers have been developed as biorecognition probes in different detection system [20, 21]. Whether A10-2 could be developed into one molecular probe to detect GP73 was further studied. The results of mFold analysis indicated that A10-2 may have a stable secondary structure because of the lower dG (−5.1). Then, the equilibrium *K*_*d*_ was calculated by ELASA assay. The *K*_*d*_ value of A10-2 was in the range of *μ*M to nM and is similar to many commercially available antibodies and others' reports [22, 23]. Interestingly, anti-GP73 antibody could block the binding of A10-2 to GP73, which might be due to the steric hindrance of the formed antigen-antibody complex. The specific binding of A10-2 to GP73 was also supported by the observation that several tumor cell lines exhibited variable expression level. Significantly, A10-2 could distinguish normal and cancerous liver tissues, where the biotin-labeled A10-2 staining was mainly located in the margin of normal liver bile duct. On the contrary, the biotin-labeled A10-2 staining was broadly distributed in the whole liver tissues of HCC patients. There are reports that GP73 was predominantly expressed by the epithelial lineage cells in normal liver and extensively expressed in HCC hepatocytes [24, 25]. The pattern of biotin-labeled A10-2 staining in liver tissues was consistent with the distribution of GP73 protein in liver tissues. These results demonstrate that A10-2 could specially recognize GP73 and distinguish HCC tissues from normal live specimens.

In summary, we successfully obtained one ssDNA aptamer that can specifically bind HCC marker protein GP73 by SELEX technique. The identified aptamer A10-2 could specifically bind GP73 with high binding capacity. Moreover, the aptamer A10-2 might be developed into one molecular probe to detect HCC from normal liver specimens.
